# Effects of Spring Rest Grazing on Underground Bud Bank in Alpine Meadow of Tibetan Plateau Before Critical Period of Soil Thawing

**DOI:** 10.3390/plants15162449

**Published:** 2026-08-12

**Authors:** Meimei Bai, Shaochong Wei, Changlin Xu, Xiaojun Yu

**Affiliations:** Key Laboratory of Grassland Ecosystem, Ministry of Education/Sino, U.S. Center for Grazing Land, College of Grassland Science, Gansu Agricultural University, Lanzhou 730070, China; 1073323010026@st.gsau.edu.cn (M.B.); weisc@st.gsau.edu.cn (S.W.); xucl@gsau.edu.cn (C.X.)

**Keywords:** Tibetan Plateau, alpine meadow, spring rest grazing, underground bud bank density, bud density model fitting

## Abstract

The bud bank is the reproductive body bank of grassland plants, which plays a key role in population maintenance and renewal. To study the effects of rest grazing on underground bud bank density, the changes in bud bank density were clarified to provide reference for grassland grazing management. In this experiment, according to the soil thawing and forage grass regreening in spring, the time when the cold-season pasture began to rest was determined. The period of grass withering is the end time of rest grazing. Different grazing rest periods are as follows: critical period of soil thawing–plant withering (RP1), early period of grass revival–plant withering (RP2) and period of local traditional rest grazing–plant withering (RP3). We studied the effects of different periods of rest grazing on the density and composition of underground bud banks. The results showed that the total density of underground bud banks increased by 33%, and the density of tiller buds, rhizome buds and root tiller buds increased by 9~62% compared with RP3. The proportion of different buds in different plots was tiller bud > rhizome bud > root tiller bud > bulb bud. Compared with RP3, the tiller bud density in RP1 increased by 24%. In RP1, the aboveground biomass and branch density of plants in RP1 were significantly higher than those in RP3 (*p* < 0.05), which were 56% and 29% higher than those in RP3, respectively. Finally, it is concluded that grazing rest before soil thawing in spring alpine meadow can promote the increase in plant underground bud bank density and the accumulation of aboveground biomass.

## 1. Introduction

China is rich in natural grassland types, which account for about 41.7% of the land area, and it is the second largest grassland resource country in the world [1]. Among them, the alpine meadow area is 1.6 × 10^6^ km^2^ [2], accounting for more than 50% of the Tibetan Plateau area, which is of special significance for maintaining regional and even global ecological security [3]. However, due to global climate change and anthropogenic factors, alpine meadows in some regions have experienced varying degrees of degradation. This not only hinders the development of local animal husbandry but also poses a serious threat to regional ecological security. Currently, among the commonly used measures for grassland restoration, grazing management measures are relatively simple and convenient to implement, making appropriate grazing practices easier to promote in pastoral areas. The grass regreening period and fruiting period are the main periods for the implementation of grassland rest grazing, so as to ensure the smooth greening of the grassland, vegetation recovery and the supply of livestock forage for the following year. However, due to global warming, the thawing of alpine meadow soil begins earlier in early spring. Moreover, under freeze–thaw cycles, the soil structure is prone to damage. If the traditional rest-grazing schedule is followed, continued grazing by yaks and Tibetan sheep after the soil thaws will trample and peel off the sod layer. This damages the plant roots and buds in the shallow soil, affecting subsequent plant regreening, growth, development, and reproduction [4]. At the same time, it destroys the biological soil crust and leads to the erosion of the surface soil. This tends to cause soil nutrient leaching and undermines the stability of the grassland ecosystem. Therefore, accurately determining the spring grazing cessation period is of great significance for reducing soil nutrient loss, enhancing grassland productivity and ensuring the plant regreens smoothly.

The plant propagule bank is the core of maintaining the continuity of grassland plant populations and the storage effect of plant species [5]. The underground bud bank is one of the important components of the plant propagule bank. As an important part of the plant reproduction bank, the underground bud bank occupies a key position, covering all buds of underground organs and aboveground organs [6], which affects the potential composition and biomass of grassland plant communities to a certain extent. Plant underground buds are attached to the underground organs of the plant and are directly connected to the parent plant. After germination, they can not only use the nutrients stored in the attached plant underground organs, but also share the photosynthetic products produced by the aerial parts of the parent plant [7]. And they quickly become new plant bodies or aboveground components of the plant through vegetative propagation or vegetative growth. The underground bud bank of a plant is the basis of plant regeneration [8]. Therefore, plants that reproduce on the underground bud bank often have a greater advantage in natural grassland [9]. Grassland underground bud banks are very important for grassland ecosystems. Bud banks play a key role in regulating plant population and community composition and structure, as well as regulating ecosystem functions to cope with environmental changes and disturbances [10]. Under environmental changes and human disturbance, the density and composition of underground bud banks can determine plant community structure and dynamics [11]. If conditions permit, long-term monitoring can be carried out to predict and explain changes in ecosystem functions. Currently, extensive research has been conducted on grassland belowground bud banks. Climate, soil nutrients and animal disturbances will affect the underground bud bank. The effects of precipitation and temperature changes on grassland are different due to different regions and grassland types. The bud diversities of temperate grasslands and alpine grasslands [12,13] were positively correlated with precipitation. Temperature has little effect on alpine grassland and has a negative impact on the density of underground buds in Arctic and temperate grasslands [14,15,16]. The underground bud banks’ size and distribution are also restricted by soil nutrient supply [17]. When the soil nutrient is sufficient, the type of bud will be more uniform and richer [18]. For example, in temperate grasslands, increased soil organic carbon promotes the growth of underground buds [19]. Studies on alpine meadows have shown that moderate disturbance by herbivores increases the density of bud banks [14]. Because the underground bud bank is sensitive to grazing intensity, rest grazing time, fertilization and other management measures, it is often used as an important basis for measuring the health status and management effect of grassland. Reasonable management (such as timely rest grazing in spring) can promote the development of underground bud banks and enhance the sustainable utilization of grassland. However, there is no report on how the grazing rest measures affect the underground bud bank of alpine meadow.

In this experiment, the local spring traditional rest grazing time of alpine meadow in Gansu Province was advanced to the critical node of soil thawing, and it was assumed that the underground part of the plant was less stressed in the critical period of soil thawing; this was conducive to the development of underground bud banks and the increase in aboveground branches and ultimately promoted the accumulation of aboveground biomass. In this experiment, three rest grazing treatments were set up: the critical period of soil thawing, the early stage of grass regreening, and the traditional rest grazing period. This study explored the effects of rest grazing on the density of underground bud banks and the proportion of various buds at different time points in spring, and selected the most suitable rest grazing period for the proliferation of underground bud banks and the vegetation, in order to serve the sustainable utilization and scientific management of alpine meadow.

## 2. Results

### 2.1. Effects of Rest Grazing in Different Periods of Spring on the Density and Proportion of Underground Bud Bank in Alpine Meadow

**Figure 1:**
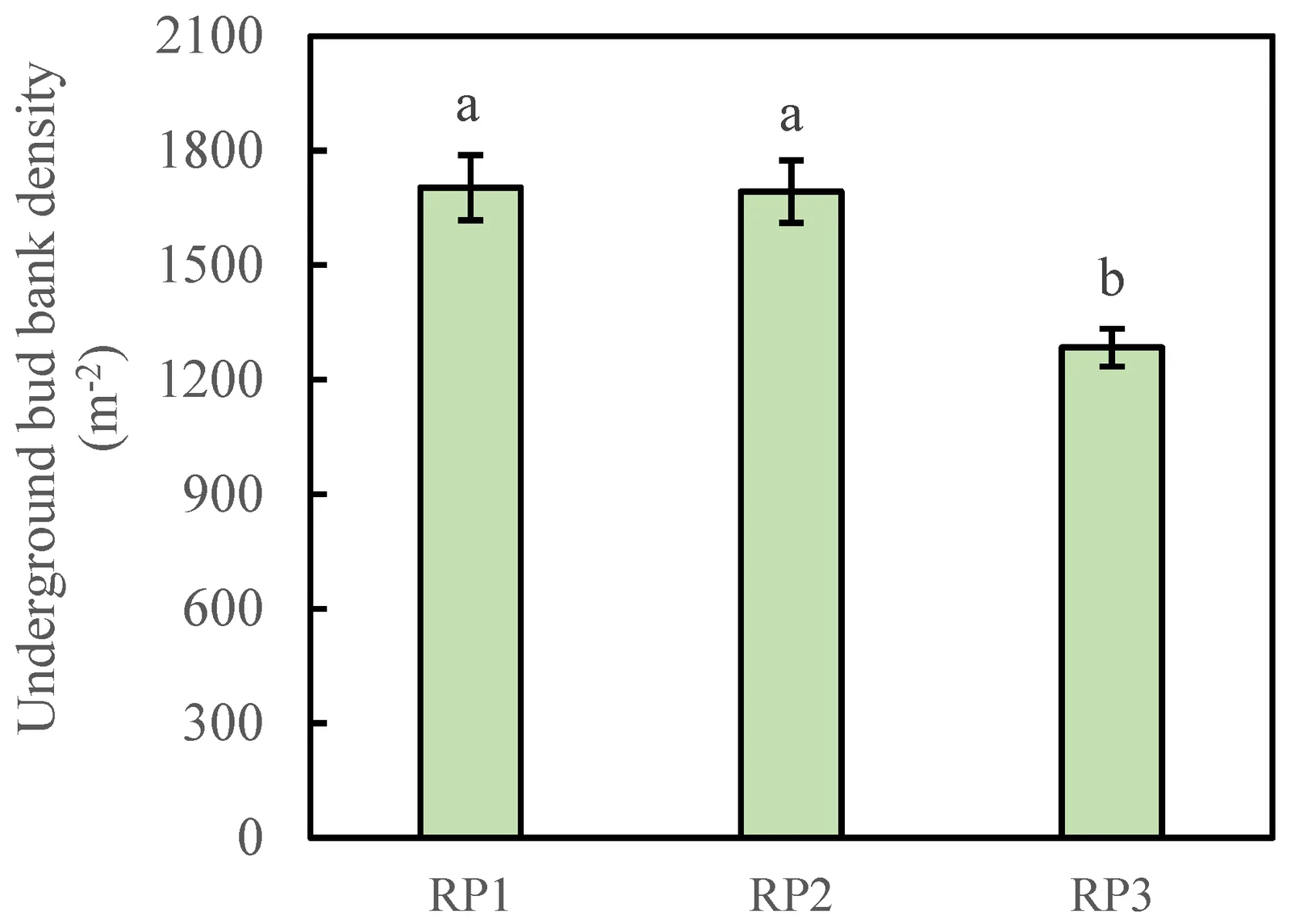
Underground bud bank density under different periods of rest grazing. RP1, RP2 and RP3 represent different fallow treatments. The fallow periods for these treatments are the critical period of soil thawing–plant withering (RP1), the early period of grass revival–plant withering (RP2), and the period of local traditional rest grazing–plant withering (RP3). Differences among multiple groups were evaluated by one-way analysis of variance (ANOVA), with a significance threshold of α = 0.05. When significant differences were detected (*p* < 0.05), Duncan’s multiple range test was conducted for post hoc comparisons. Different lowercase letters indicate significant differences between treatments (*p* < 0.05), whereas the same letters indicate no significant difference.

From Figure 1, the underground bud banks’ total density of RP1, RP2 and RP3 was 1702.94 m^−2^, 1692.70 m^−2^, and 1284.27 m^−2^, respectively. Compared with RP3, the total density of underground bud banks in RP1 and RP2 was significantly higher (*p* < 0.05), with increases of 32.60% and 31.80% respectively.

**Figure 2:**
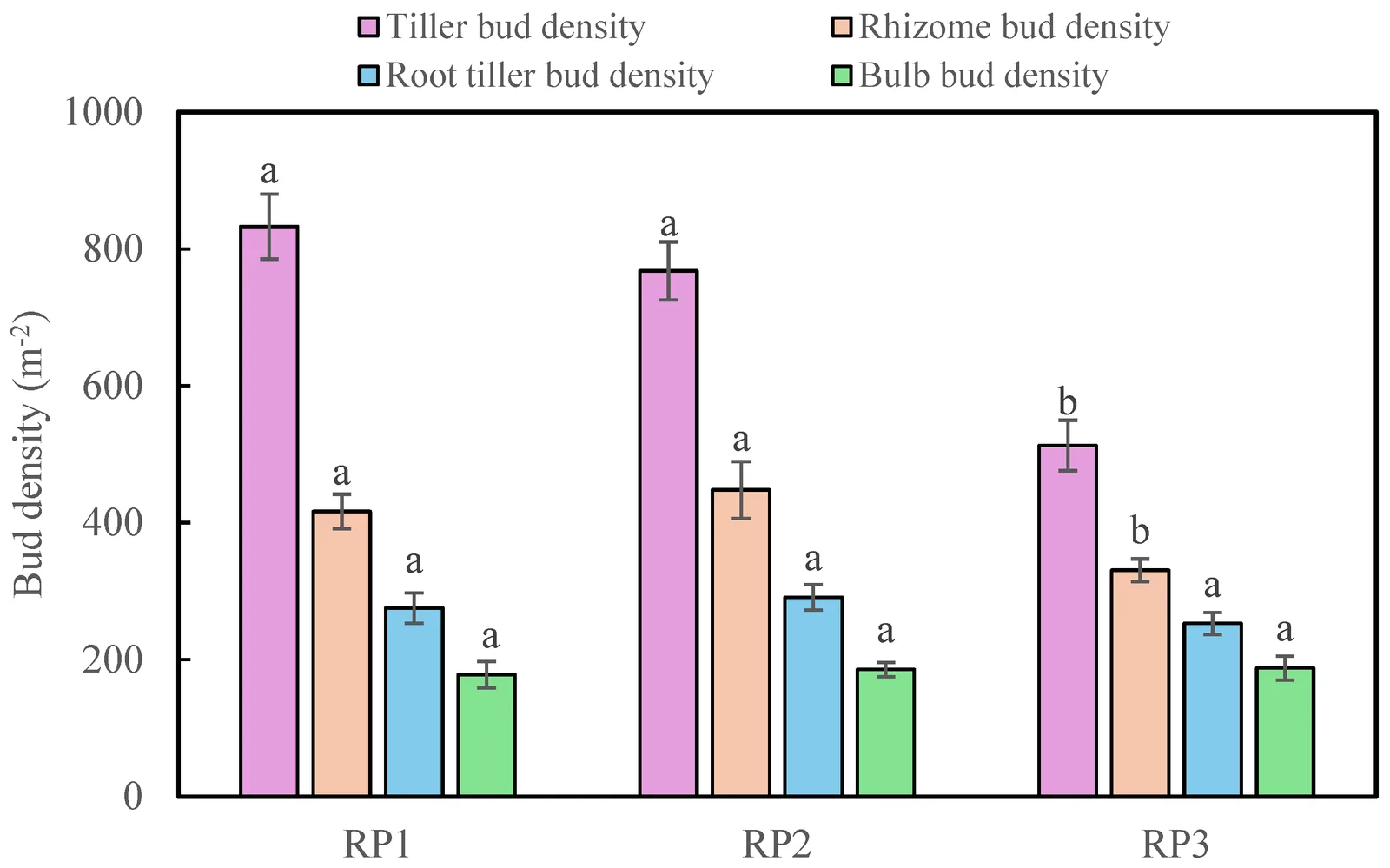
Different bud densities under different periods of rest grazing. Different lowercase letters indicate significant differences between treatments (*p* < 0.05), whereas the same letters indicate no significant difference.

Figure 2 shows that the density of different buds in each treatment was tiller bud > rhizome bud > root tiller bud > bulb bud. The tiller bud densities of RP1 and RP2 were 832.90 m^−2^ and 767.94 m^−2^, respectively, which were significantly higher than those of RP3 (*p* < 0.05), and were 62.38% and 49.72% higher than those of RP3, respectively. The rhizome bud density of RP3 was significantly lower than that of RP1 and RP2, which was 20.61% and 26.18% lower than that of RP1 and RP2, respectively.

**Figure 3:**
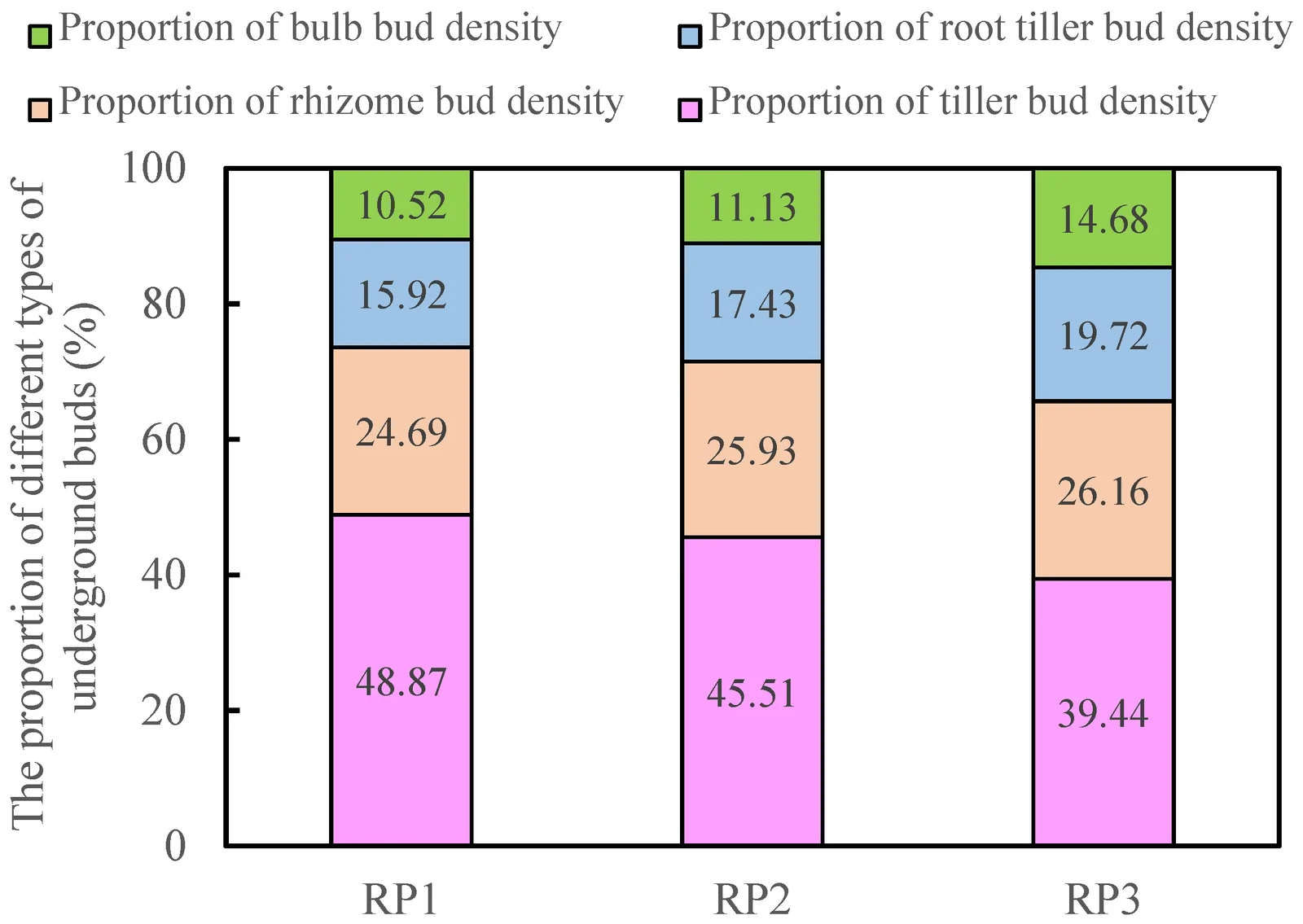
The proportion of different types of underground buds under different periods of rest grazing.

From Figure 3, it can be seen that the density proportion of different buds in each treatment was tiller bud > rhizome bud > root tiller bud > bulb bud. The proportion of tiller buds was RP1 > RP2 > RP3, and the tiller bud density of RP3 was 11.96% and 13.34% lower than that of RP1 and RP2, respectively. The order of density proportion of rhizome bud, root tiller bud and bulb bud was opposite to that of tiller bud, which was RP3 > RP2 > RP1. The density of rhizome buds of RP3 was 5.95% higher than that of RP1; the density of root tiller buds was 23.87% and 13.14% higher than that of RP1 and RP2 respectively, and the density of bulb buds was 39.54% and 31.90% higher than that of RP1 and RP2 respectively.

### 2.2. Effects of Rest Grazing at Different Stages on Aboveground Biomass, Branch Density and Meristem Limitation Index of Plants

**Figure 4:**
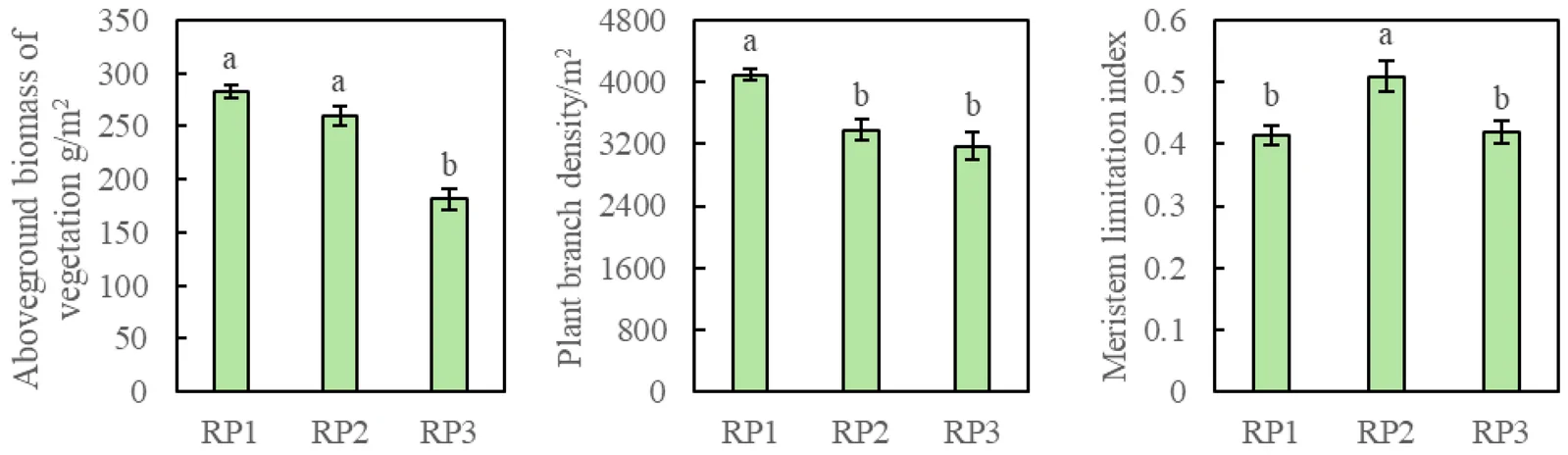
Plant branch density, aboveground biomass of vegetation, and meristem limitation index of plants under different periods of rest grazing. Different lowercase letters indicate significant differences between treatments (*p* < 0.05), whereas the same letters indicate no significant difference.

As shown in Figure 4, the aboveground biomass of RP1 and RP2 was significantly higher than that of RP3 (*p* < 0.05), being 55.76% and 43.35% higher than that of RP3, respectively. The plant branch density of RP1 was significantly higher than that of RP2 and RP3 (*p* < 0.05), which was 21.03% and 29.08% higher than that of RP2 and RP3, respectively. There was no significant difference between RP2 and RP3. The meristem restriction coefficient of RP2 was significantly higher than that of RP1 and RP3, which was 22.99% and 21.32% higher than that of RP1 and RP3, respectively.

### 2.3. Variability Analysis of Plant Underground Bud Bank and Related Indexes

The variation degree of underground bud bank and related indexes differed with different rest grazing times (Table 1). The variation coefficient of total density of underground bud bank ranged from 17.19% to 22.32%, the variation coefficient of different bud density ranged from 22.42% to 48.80%, the variation coefficient of aboveground biomass ranged from 10.08% to 24.38%, the variation coefficient of plant ramet density ranged from 8.11% to 25.62%, and the variation coefficient of meristem restriction coefficient ranged from 17.45% to 22.15%. By comparing the coefficient of variation of underground bud banks and related indicators, it is shown that with the change in the start time of rest grazing, the degree of variation in most indicators in various plots reaches more than 20%.

### 2.4. Correlation Analysis of Plant Underground Bud Bank and Related Indexes

Correlation analysis (Table 2) showed that the aboveground biomass of vegetation was significantly positively correlated with the total density of underground bud banks (*p* < 0.01) (the correlation coefficient is 0.643) and positively correlated with the density of all kinds of buds, among which the densities of tiller buds, rhizome buds, and root tiller buds reached a very significant level (*p* < 0.01); the correlation coefficients were 0.616, 0.418, and 0.348, respectively.

The density of plant ramets was positively correlated with the density of various buds, among which the density of tiller buds and rhizome buds was significantly correlated (*p* < 0.01), and the density of ramets was significantly positively correlated with the aboveground biomass (*p* < 0.01). The meristem restriction index was significantly positively correlated with bud bank density (*p* < 0.01) and significantly negatively correlated with plant branch density (*p* < 0.01).

### 2.5. Vegetation Biomass, Underground Bud Density and Plant Ramet Density Model Fitting

**Figure 5:**
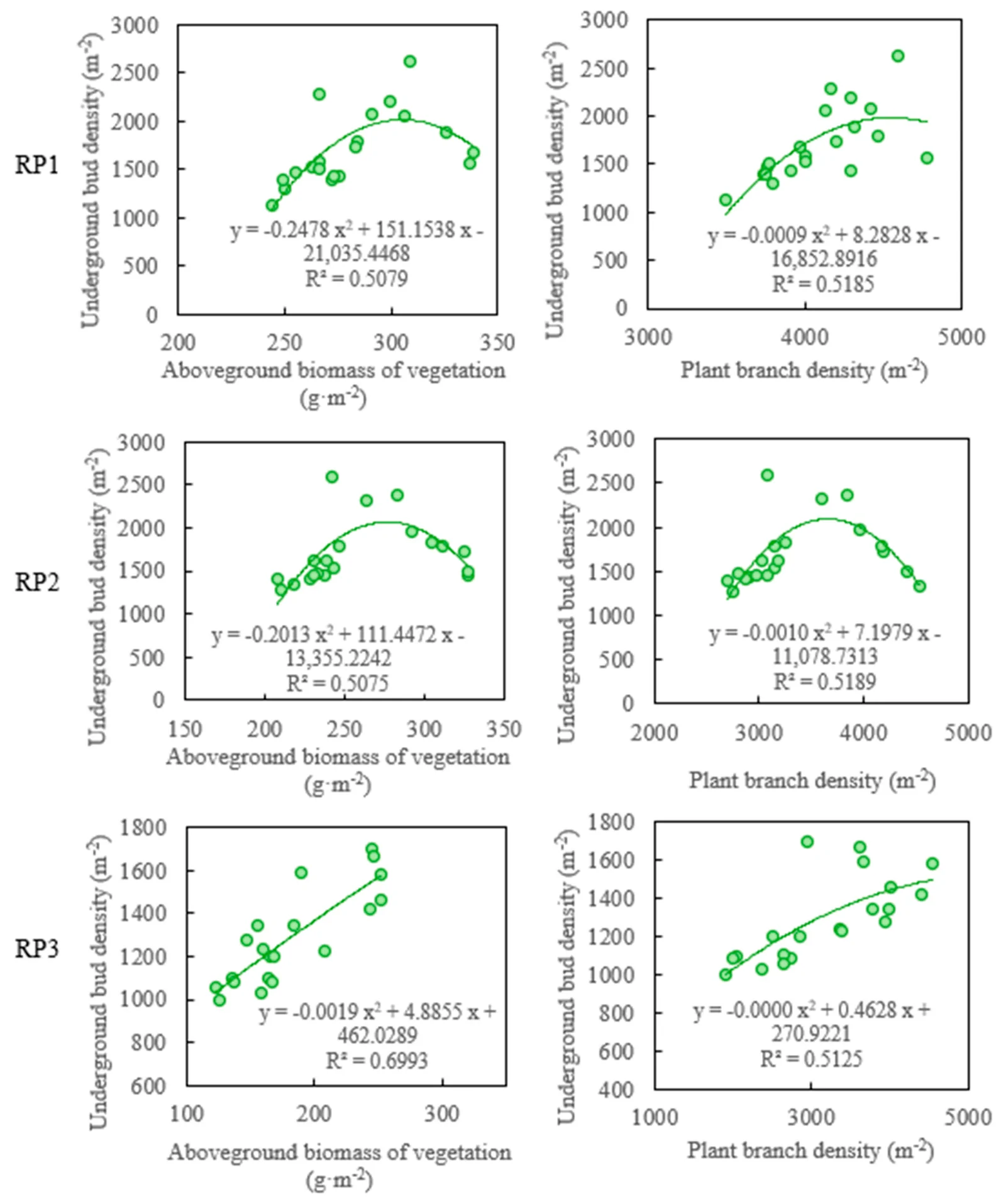
The optimal fitting equation for underground bud density with vegetation aboveground biomass and plant branch density.

The relationship between the total density of the underground bud bank and the aboveground biomass of vegetation and the density of plant branches in each treatment was fitted by various functional models, and the polynomial function provided the best fit (Table 3). The polynomial function had the best fitting degree. In the process of fitting the total density of bud bank and the density of plant ramets, the R^2^ of RP1, RP2 and RP3 reached 0.5185, 0.5189 and 0.5125 respectively. The R^2^ of RP1, RP2 and RP3 reached 0.5079, 0.5075 and 0.6993 respectively in the fitting process of total bud density and plant aboveground biomass (Figure 5).

**Table 3:** Fitting analysis of underground bud density (D), aboveground vegetation biomass (M) and plant branch density (P).

	D + P					D + M			
	Equation	a	b	c	R2	a	b	c	R2
RP1	D = a × M + b	0.7545	−1386.601	—	0.4340	6.6503	−177.0221	—	0.2483
	D = a × Mb	0.0003	1.8519	—	0.4191	1.7256	1.2186	—	0.2431
	D = a + b × In(M)	−24,542	3156.6023	—	0.4494	−9651.5318	2013.3252	—	0.2717
	D = a × eb × M	273.5425	0.0004	—	0.3996	533.3093	0.0040	—	0.2200
	D = a × M2 + b × M + c	−0.0009	8.2828	−16,853	0.5185	−0.2478	151.15	−21,035	0.5079
RP2	D = a × M + b	0.1366	1230.7233	—	0.0486	2.3865	1071.8123	—	0.0711
	D= a × Mb	104.8796	0.3404	—	0.0612	132.0943	0.4560	—	0.0819
	D = a + b × In(M)	−2941.1739	571.1701	—	0.0682	−2322.6826	723.4865	—	0.0915
	D = a × eb × M	1258.1408	0.0001	—	0.0434	1116.0153	0.0015	—	0.0634
	D = a × M2 + b × M + c	−0.0010	7.1979	−11,079	0.5189	−0.2013	111.4472	−13,355.2242	0.5075
RP3	D = a × M + b	0.1921	675.0314	—	0.4998	4.1721	527.1175	—	0.6991
	D= a × Mb	29.3387	0.4690	—	0.5069	60.0454	0.5894	—	0.6988
	D = a + b × In(M)	−3442.8892	588.7436	—	5094	−2718.8674	773.7227	—	0.6941
	D = a × eb × M	780.0912	0.0002	—	0.4894	713.4070	0.0032	—	0.6961
	D = a × M2 + b × M + c	−0.0001	0.4628	270.9221	0.5125	−0.0019	4.8855	462.0289	0.6993

## 3. Discussion

### 3.1. The Density of Underground Bud Bank and the Proportion of Various Buds Under Different Periods of Rest Grazing

Disturbances in alpine meadows can exert complex influences on population regeneration and even the composition of plant communities through multiple pathways, including alterations in soil physicochemical properties, the size and composition of propagule banks, seedling survival and establishment, as well as biotic interactions. As a critical propagule bank for the maintenance and regeneration of grassland plant populations, the bud bank can be regarded as a key driving force of vegetation renewal and community dynamics in grasslands. Its contribution to population regeneration largely determines plant community structure and ecosystem functioning [14]. In addition, the underground bud bank is also of great significance for regulating the response of grassland ecosystem function and plant community structure to environmental changes [20]. The underground bud bank (such as buds on rhizomes, tillering nodes, bulbs, etc.) is the core source of vegetative reproduction of grassland plants. Compared with seed reproduction, the regeneration of underground bud banks is more stable, especially in grassland ecosystems with strong environmental stresses such as cold and drought. Its inherent concealment can avoid most external stress, thus ensuring the interannual continuation of the plant population and the relative stability of the community structure. The size, species composition and spatial distribution of underground bud banks directly affect the number, density and growth potential of aboveground branches, and then determine the species composition, structural characteristics and aboveground biomass of grassland vegetation communities.

Combined with the results of this experiment, early rest grazing in spring in alpine meadows can significantly affect the density of the underground bud bank. In this experiment, the total density of plant underground bud bank was ranked as RP1 > RP2 > RP3. The results showed that the total density of plant underground bud bank increased gradually with the advance of rest grazing, and the total density of bud bank in RP1 was 33% higher than that in RP3. When the height of the dominant herbage reached 5 cm, the grazing was stopped in the traditional rest grazing plot. At this time, the plants were trampled and fed by livestock for a long time. Due to the influence of global warming, the soil thawing time of the alpine meadow was advanced. In the grazing process, the shallow buds of the soil in the traditional rest grazing plot were more vulnerable to livestock trampling, which directly affected the growth of the underground bud bank of plants in spring. At the same time, the nutrients stored in the underground part can be supplied to the repair of damaged parts, which increases the energy consumption of plants and also has a negative impact on the growth of underground buds in the later stage. Another study pointed out that when the spring rest grazing time of the alpine meadow was advanced to the critical period of soil thawing, the ammonium nitrogen, soil moisture, organic carbon, available phosphorus and active organic carbon content were maintained at a high level [21]. This higher soil nutrient condition plays a positive role in promoting the growth of plant propagules. The number of tillers of *Elymus nutans* and *Carex alatauensis* in the experimental plots increased with the advance of rest grazing in spring [22,23], which was confirmed by the changes in tiller buds in the bud bank. In this experiment, the order of density and proportion of various buds in the underground bud bank of plants was tiller bud > rhizome bud > root tiller bud > bulb bud. The change in rest grazing time had no significant effect on the composition of the underground bud bank. In this experimental plot, *Carex capillifolia* was the constructive species, and *Bistorta vivipara*, *C. alatauensis*, *E. nutans* were the dominant species. The importance of *C. capillifolia* was higher than that of other plants [24], while *C. capillifolia*, *C. alatauensis*, *E. nutans* had tiller buds and rhizome buds, and other gramineous and leguminous plants had only tiller buds, so the plot had more tiller buds than rhizome buds. There are few plants with root and tiller buds in the experimental plot, such as *Artemisia smithii*, *Potentilla bifurca*, *Anaphalis lactea*, *Taraxacum mongolicum*, and *Galium boreale*. These plants account for a relatively low proportion in the whole plot, and only *Allium cyaneum* has bulb buds in the plot. The vegetation composition of the grassland determines the density and composition of the underground bud bank. The density and composition of the underground bud bank will affect the subsequent vegetation composition of the grassland. The plant propagule and the plant body interact with each other, and finally form the vegetation characteristics of the grassland, which are more stable under certain environmental conditions.

### 3.2. The Correlation of Plant Underground Bud Bank Density, Aboveground Biomass and Branch Density Under Different Periods of Rest Grazing

Under the combined influence of environmental factors and human factors such as climate change and overgrazing, the survival strategy and reproduction strategy of constructive species and key species in alpine meadow [25,26], plant community composition and structure, system resilience and stability maintenance ability have changed to varying degrees [27]. The experimental site is a typical alpine *Kobresia* meadow. The double-layer structure of Cyperaceae and Gramineae ensures the high production service and system anti-interference ability of the grassland [28]. Due to its unique vegetation composition, it is determined that the alpine meadow plants are dominated by asexual reproduction. As the main body of asexual reproduction, the bud bank is of great significance to maintain the number of plant populations, the spatial distribution of species, and the stability of the community in the degraded alpine *Kobresia* meadow.

A plant branch population is a collection of all ramets produced by asexual reproduction of the same genet. Its structure and dynamic changes not only affect the individual resource acquisition and reproduction strategy of plants but also play a key role in community construction and ecosystem stability maintenance by regulating intraspecific and interspecific competition, community composition and ecosystem function [29]. The underground bud bank is the “reserve bank” and “potential driving force” of the aboveground population (branches density and biomass). Its size and composition largely determine the regeneration ability and productivity level of aboveground vegetation. The underground bud bank is the “reserve bank” and “potential driving force” of the aboveground population (branches density and biomass), and its size and composition largely determine the regeneration ability and productivity level of the aboveground vegetation. All kinds of buds in the underground bud bank are the source of aboveground branches (new plants). Studies have confirmed that different types of buds develop into different aboveground ramets. For example, in *Leymus chinensis* populations, tiller node buds contribute about 79% of the aboveground ramets and are the main force for population maintenance [30]. In the typical steppe of the Loess Plateau, there was also a significant positive correlation between the density of grass buds and aboveground biomass [31]. The results of this study also showed that the density of underground bud banks was significantly positively correlated with the aboveground biomass of vegetation and the density of plant branches, which was consistent with previous studies. The growth of underground bud banks of grassland plants was closely related to the regeneration of plants after the renewal, loss or interference of new shoots on the ground. Bud banks have a potential stabilizing effect on plant population dynamics [6]. Detailed plant population statistics have shown that the smooth development of seeds into seedlings is rare and accidental. In contrast, the contribution of buds to the annual regeneration of aboveground populations is more significant, sometimes more than ten times that of seeds [32,33,34]. This study reveals that the aboveground biomass of vegetation had the strongest correlation with tiller buds, which was mutually confirmed by the large proportion of plants with tiller buds such as Cyperaceae and Gramineae in the plot.

The climate of the Tibetan Plateau has the characteristics of low temperature and strong wind. The climate and geographical location lead to a short growth period of plants. Tillering is an efficient, energy-saving and safe reproductive and survival strategy formed under this extreme environmental pressure. Tillering buds are usually located on the surface or underground, and are tightly wrapped by litter, soil and leaf sheath, which can effectively protect key bud points and make them more likely to survive than single-stem plants with exposed apical buds. On the other hand, a plant usually has multiple tiller buds. Even if the aboveground part is seriously damaged by livestock feeding and trampling, or low temperature, as long as the tiller buds survive, they can germinate quickly when the conditions are suitable, rebuild the plant, greatly improve the survival rate, and play a role in dispersing risks. Through tillering, plants can form low and dense grasses. This structure can effectively reduce the wind speed on the surface, reduce water evaporation, and form a relatively stable, humid and warm microenvironment inside the grasses, which is conducive to the growth of plants’ own branches. This absolute evolutionary advantage ultimately makes Cyperaceae, Gramineae and other plants become dominant species in alpine regions. The aboveground ramets of plants are the products of the germination and growth of the underground bud bank. The underground bud bank is the “source” of the aboveground ramets of plants, and the aboveground ramets are the “sink” of the underground bud bank. A higher branch density indicates a larger bud bank density and more germination units. When the aboveground branches of plants are damaged by grazing, mowing, etc., the underground bud bank will be activated rapidly, and new ramets will germinate to supplement the aboveground part and maintain the stability of the population. Rest grazing during the critical period of soil thawing avoids the trampling of plant buds by livestock. The nutrients stored in the underground part preferentially meet the needs of plant regreening and subsequent growth, avoiding the use of energy for the recovery of damaged parts. Therefore, more nutrients are used for the growth of the aboveground parts, and the aboveground vegetation has more ramets and biomass, producing more photosynthetic products and feeding back to the bud bank. In the early stage of population establishment (when the branch density is low), plants will give priority to investing in the production of extended buds such as rhizome nodal buds to quickly occupy new space. As the population develops (the branch density becomes higher), plants will use more resources to produce tiller nodal buds to consolidate the territory and increase the population density, so as to occupy and maintain the space. The underground bud bank determines the number (density) of aboveground branches through germination, which in turn affects the accumulation of aboveground biomass. At the same time, the density state of the aboveground population will in turn affect the investment strategy of the plant in the underground bud bank.

## 4. Materials and Methods

### 4.1. Experimental Plot Overview

The experiment was conducted in a cold-season pasture located at the eastern end of the Qilian Mountains (37°40′ N, 102°32′ E) on the Tibetan Plateau, adjacent to the Tianzhu Alpine Grassland Experimental Station of Gansu Agricultural University, and administratively affiliated with Wuwei City, Gansu Province. The altitude of the test site is 2960 m, indicating a continental plateau monsoon climate. The cold and warm seasons are distinct: the warm season is from June to September, and the cold season is from October to May of the next year [23]. The soil is subalpine meadow soil (USDA Soil Taxonomy: Cryrendolls) [35], the grassland is alpine meadow. The constructive species is *C*. *capillifolia*, and the dominant species are *E*. *nutans*, *B*. *vivipara*, *C*. *alatauensis* and so on.

### 4.2. Experimental Design

In 2018, plots with uniform vegetation were selected, and fences were used to separate the sample plots required for the grazing test. The rest grazing time was determined based on the depth of soil thaw, the regreening status of forage, and the local traditional rest grazing. The soil thawing depth was measured by the average value of the two measurements at 9:00 a.m. and 2:30 p.m. The end time of rest grazing was uniformly set in the withering period of forage grass, and each treatment plot was recorded as RP1, RP2 and RP3, respectively. Considering that grazing has no significant impact on frozen soil and withered vegetation during winter, grazing was initiated on 1 March. Table 1 lists the soil conditions, plant conditions and the number of grazing livestock in each grazing sample plot. The plot area is calculated and fenced according to the 80% utilization rate of forage grass.

(1) $$GA=\frac{(A-F)\times D\times N}{Y\times U}$$

In the formula, GA is the grassland area/m^2^; A is the total amount of grass required for livestock in a day (yak: 5.8 kg hay, Tibetan sheep: 1.7 kg hay [36]); F is the supplementary feeding grass quantity for one livestock per day (according to the field survey, Tibetan sheep: 0.22 kg hay, yak: 1.23 kg hay); D is the grazing days; N is the number of livestock grazing (Table 4); Y is the grass yield of the sample plot (2895 kg/hm^2^); and U is the pasture utilization (80%).

### 4.3. Experimental Methods

In August 2025, evenly distributed points were randomly selected from each plot for destructive sampling, and 20 samples were taken from each sample plot. The size of the sampling block is 20 cm (width) × 20 cm (length) × 20 cm (depth). During the sampling process, the underground part and the aboveground part of the plant were dug together, and the soil of the plant roots was carefully removed to ensure the integrity of the underground and aboveground parts of the plant [37]. Based on previous studies [38] and vegetation survey, we divided the underground bud banks into four types: tiller buds (mainly occur on the unelongated tillering nodes of plant stems, such as Gramineae, Cyperaceae and other plants), rhizome buds (buds growing at underground rhizome nodes and tops), root tiller buds (adventitious buds produced by plant roots and buds growing at short rhizomes on the surface) and bulb buds (buds wrapped by bulbs at the base of bulbous species). In this study, we only counted fresh intact buds; dead or significantly damaged buds (such as buds killed by pathogens) were not counted; and damaged buds at sampling were also excluded [39]. The number of plant branches in the aboveground part of the excavated soil was counted and then dried and the biomass was weighed. The “meristem limitation index” (MLI) was used to characterize the association between underground bud banks and aboveground plant populations. The MLI is the ratio of the density of underground bud banks to the density of aboveground ramets in the sample. The calculation formula is as follows [40]:

(2) Meristem limitation index (MLI) = bud density/branch density

### 4.4. Data Analysis

In this experiment, Origin 2021, SPSS 19.0, and Microsoft Excel 2017 software were used for data collation, analysis and drawing. One-way analysis of variance was performed on plant branch density, aboveground biomass of vegetation, plant meristem limitation index, various bud densities, and total bud density. Before the analysis, the normality and homogeneity of variance of the data were tested. Fisher’s Least Significant Difference (LSD) test and Duncan’s multiple range test were used as post hoc tests.

Basic model construction: The underground bud density was fitted with the aboveground biomass and plant branch density, and equations in the form of logarithmic, exponential, linear, polynomial and power functions were adopted to establish the correlations among the three variables, and the model with the best fitting effect was selected.

## 5. Conclusions

This study focused on the alpine meadow at the eastern end of the Tibetan Plateau and analyzed the effects of different periods of rest grazing on the density and structure of underground bud banks. Rest grazing began in the critical period of soil thawing, which increased the total density of the underground bud bank, increased the density of tiller buds, rhizome buds and root tiller buds, and increased the proportion of tiller bud density. With the advance of rest grazing, the aboveground vegetation biomass and plant ramet density gradually increased. The effect of rest grazing in different periods on the density of different types of buds is inconsistent. Rest grazing in advance of the critical period of soil thawing has a greater effect on the density of tiller buds and has a smaller effect on the density of rhizome buds and root tiller buds. In general, rest grazing on alpine meadow from the critical period before soil thawing to the yellowing stage of forage grass can promote the increase in total density of the underground bud bank of vegetation, thus promoting the accumulation of aboveground biomass of plants.

## Figures and Tables

**Table 1:** Variation analysis of plant underground bud bank and its related indexes.

Treatments	Underground Bud Density	Tiller Bud Density	Rhizome Bud Density	Root Tiller Bud Density	Bulb Bud Density	Aboveground Biomass of Vegetation	Plant Branch Density	Meristem Limitation Index
RP1	22.32	25.44	27.11	36.11	48.80	10.08	8.11	17.45
RP2	21.57	24.75	41.58	28.65	25.13	15.68	17.42	22.15
RP3	17.19	32.11	22.42	28.61	41.32	24.38	25.62	18.56

(Unit: %).

**Table 2:** Correlation analysis of plant underground bud bank and its related indexes.

	Underground Bud Density	Tiller Bud Density	Rhizome Bud Density	Root Tiller Bud Density	Bulb Bud Density	Aboveground Biomass of Vegetation	Plant Branch Density	Meristem Limitation Index
Underground bud density	1.000							
Tiller bud density	0.858 **	1.000						
Rhizome bud density	0.726 **	0.397 **	1.000					
Root tiller bud density	0.639 **	0.360 **	0.419 **	1.000				
Bulb bud density	0.324 *	0.085	0.104	0.202	1.000			
Aboveground biomass of vegetation	0.643 **	0.616 **	0.418 **	0.348 **	0.168	1.000		
Plant branch density	0.502 **	0.455 **	0.275 *	0.385 **	0.181	0.649 **	1.000	
Meristem limitation index	0.582 **	0.453 **	0.534 **	0.309 *	0.193	0.0830	−0.398 **	1.000

** At the 0.01 level (two-tailed), the correlation is extremely significant. * At the 0.05 level (two-tailed), the correlation is significant.

**Table 4:** Rest grazing experiment design.

Plots	Rest Grazing Treatments	Conditions	Specific Grazing Rest Time	Number of Grazing Livestock (Adult)	Sample Area/m^2^
RP1	Critical period of soil thawing–plant withering	Soil thawing depth > 0.9 cm	19 March~28 February of the following year	4 Yaks + 4 Tibetan Sheep	1881
RP2	Early period of grass revival–plant withering	Re-greening coverage reached 30–40%	16 April~28 February of the following year	4 Yaks + 4 Tibetan Sheep	4807
RP3	Period of local traditional rest grazing–plant withering	Dominant plant height was about 5 cm	21 May~28 February of the following year	16 Yaks + 16 Tibetan Sheep	33,855

## Data Availability

With regard to the request for raw data, we would like to clarify that although the data is not stored in a public repository, we have a strict data availability policy. Since the data used in this paper is also a confidential project, the data of the research results will not be disclosed for the time being. The datasets used and analyzed in this study can be obtained from the corresponding authors upon reasonable request.

## References

[1] Shen H.H., Zhu Y.K., Zhao X., Geng X.Q., Gao S.Q., Fang J.Y. (2016). Analysis of current grassland resources in China. Chin. Sci. Bull..

[2] Dong S.K., Zhang Y., Shen H., Li S., Xu Y.D. (2023). Overview of the third pole’s grasslands. Grasslands on the Third Pole of the World: Structure, Function, Process, and Resilience of Social-Ecological Systems.

[3] Chen B. (2023). Regulatory Mechanisms Under Alpine Grasslands Sensitivity to Climate Change on the Qinghai-Tibetan Plateau. Master’s Thesis.

[4] Jing Y.Y., Bai M.M., Xu C.L., Wang L., Yang H., Jiang J.C., Wang H., Yu X.J. (2022). Advancing the spring rest-grazing time until the critical period when soil thaws promotes soil recovery and bacterial diversity in alpine meadows. Ecol. Indic..

[5] Klimešová J., Klimeš L. (2007). Bud banks and their role in vegetative regeneration-a literature review and proposal for simple classification and assessment. Perspect. Plant Ecol. Evol. Syst..

[6] Ott J.P., Klimešová J., Hartnett D.C. (2019). The ecology and significance of below-ground bud banks in plants. Ann. Bot..

[7] Ott J.P., Hartnett D.C. (2015). Bud-bank and tiller dynamics of co-occurring C3 caespitose grasses in mixed-grass prairie. Am. J. Bot..

[8] He K.Y., Zhou Q.P., Dang H.H., Wang X.L., He L.L., Wei X.X., Li J.Y., Wang Q., Wang J.H. (2026). Soil-mediated regulatory mechanisms of belowground bud banks in the sustainable management and ecological restoration of degraded alpine grasslands. Sustainability.

[9] Fang T., Ye D., Gao J.J., Luo F.L., Zhu Y.J., Yu F.H. (2024). Responses of belowground bud bank density of geophytes to environmental perturbations: A meta-analysis. J. Plant Ecol..

[10] Qian J.Q., Tian J.T., Zheng X.Y., Li D.M., Lubbe F.C., Liu D., Yang J.J., Cao J.R., Wang Q.B., Zhang Z.M. (2026). Litter manipulation alters the belowground bud bank and aboveground shoot density in a temperate grassland. Agric. Ecosyst. Environ..

[11] Dalgleish H.J., Kula A.R., Hartnett D.C., Sandercock B.K. (2008). Responses of two bunchgrasses to nitrogen addition in tallgrass prairie: The role of bud bank demography. Am. J. Bot..

[12] Ma Q., Qian J.Q., Shi X.Y., Qin X.P., Tian L., Liu Z.M. (2023). Temperature-related factors are the better determinants of belowground bud density, while moisture-related factors are the better determinants of belowground bud diversity at the regional scale. Land Degrad. Dev..

[13] Ding X.J., Su P.X., Zhou Z.J., Shi R. (2019). Belowground bud bank distribution and aboveground community characteristics along different moisture gradients of alpine meadow in the Zoige Plateau, China. Sustainability.

[14] Li Y., Bao G.S., Zhang P., Feng X.Y., Ma J.J., Lu H.N., Shi H.X., Wei X.X., Tang B.M., Liu K. (2023). Changes in bud bank and their correlation with plant community composition in degraded alpine meadows. Front. Plant Sci..

[15] Klimešová J., Doležal J., Prach K., Košnar J. (2012). Clonal growth forms in Arctic plants and their habitat preferences: A study from Petuniabukta, Spitsbergen. Pol. Polar Res..

[16] Guittar J., Goldberg D., Klanderud K., Telford R.J., Vandvik V. (2016). Can trait patterns along gradients predict plant community responses to climate change?. Ecology.

[17] Zhang J.F. (2017). Responses of Bud Bank Dynamics of *Nitraria tangutorum* to Nitrogen Addition. Master’s Thesis.

[18] Klimeš L. (2008). Clonal splitters and integrators in harsh environments of the Trans-Himalaya. Evol. Ecol..

[19] Jiao D.Z., Yao L., Huang Z.Y., Yang Y.F. (2015). Bud population dynamics of Phragmites australis in heterogeneous habitats of Northeast grassland, China. Chin. J. Appl. Ecol..

[20] Gupta A., Rico-Medina A., Caño-Delgado A.I. (2020). The physiology of plant responses to drought. Science.

[21] Jing Y.Y. (2023). Effects of Rest-Grazing on Soil Carbon Pool and Carbon Components in Cold Season Pasture of Alpine Meadow. Doctoral Thesis.

[22] Bai M.M., Wei K.T., Ma K.K., Xu C.L., Yu X.J. (2022). Rest grazing from the critical period of soil thawing promotes the propagation of *Kobresia humilis* in alpine meadow. Ecol. Eng..

[23] Peng Z. (2020). Effects of Rest Grazing in Different Stages of Spring on Reproduction and Root of *Kobresia capillifolia* and *Elymus nutans* in Alpine Meadow. Master’s Thesis.

[24] Ma K.K. (2023). Effects of Different Rest Grazing Periods on Soil Seed Bank, Plant Community Characteristics and Theoretical Grazing Capacity in Alpine meadow. Master’s Thesis.

[25] Shang Z.H. (2006). Studies on Soil Seed Bank and Regeneration of Degraded Alpine Grassland in the Headwaters of Yangtze and Yellow Rivers on Tibetan Plateau. Doctoral Thesis.

[26] Osland M.J., González E., Richardson C.J. (2011). Restoring diversity after cattail expansion: Disturbance, resilience and seasonality in a tropical dry wetland. Ecol. Appl..

[27] Pan Q.M., Yang Y.H., Huang J.H. (2023). Limiting factors of degraded grassland restoration in China and related basic scientific issues. Bull. Natl. Nat. Sci. Found. China.

[28] Li B.C., Cao G.M., Guo X.W., Fan B., Lan Y.T., Si M.K., Lin L. (2025). Soil seed bank characteristics of typical plant communities in alpine *Kobresia* meadows under grazing intensities. Grassl. Turf.

[29] Li S.H. (2017). Research progress of clonal plant ecology and its significance in ecological restoration. Contemp. Hortic..

[30] Zhang J.T. (2009). The Variation of All Types of Buds of Belowground Bud Bank and Their Relationship with Aboveground Shoots in *Leymus chinensis* Population. Doctoral Thesis.

[31] Bai X. (2017). Effects of Enclosure Duration on Offspring Recruitment and Bud Bank in Semiarid Steppe on the Loess Plateau. Master’s Thesis.

[32] Benson E.J., Hartnett D.C. (2006). The role of seed and vegetative reproduction in plant recruitment and demography in tallgrass prairie. Plant Ecol..

[33] Latzel V., Mihulka S., Klimešová J. (2008). Plant traits and regeneration of urban plant communities after disturbance: Does the bud bank play any role?. Appl. Veg. Sci..

[34] Vítová A., Macek P., Lepš J. (2017). Disentangling the interplay of generative and vegetative propagation among different functional groups during gap colonization in meadows. Funct. Ecol..

[35] Soil Survey Staff (2003). Keys to Soil Taxonomy.

[36] Xu P. (2000). Grassland Resource Inventory and Planning.

[37] Qian J.Q., Wang Z.W., Klimešová J., Lü X.T., Kuang W.N., Liu Z.M., Han X.G. (2017). Differences in below-ground bud bank density and composition along a climatic gradient in the temperate steppe of northern China. Ann. Bot..

[38] Qian J.Q., Wang Z.W., Klimešová J., Lü X.T., Zhang C.Y. (2021). Belowground bud bank and its relationship with aboveground vegetation under watering and nitrogen addition in temperate semiarid steppe. Ecol. Indic..

[39] Tao J., Tian J.T., Li D.M., Ma Q., Zhang Z.M., Liu W., Pan Q.M., Qian J.Q. (2025). Effects of fertilization timing on belowground bud bank and its relationship with vegetation of degraded meadow steppe. Chin. J. Ecol..

[40] Benson E.J., Hartnett D.C., Mann K.H. (2004). Belowground bud banks and meristem limitation in tallgrass prairie plant populations. Am. J. Bot..

